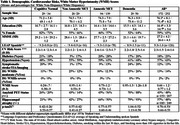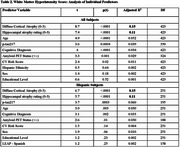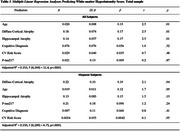# White matter hyperintensities among Hispanic and non‐Hispanic Whites: the relevance of demographic, biological and non‐biological variables

**DOI:** 10.1002/alz70856_101380

**Published:** 2025-12-25

**Authors:** Monica Rosselli, Warren W Barker, Merike Lang, Alicia Goytizolo, Breton M. Asken, David A. Loewenstein, Stephen A. Coombes, Melissa J. Armstrong, Idaly Velez‐Uribe, Jacob Fiala, Michael Marsiske, Glenn E. Smith, Ranjan Duara

**Affiliations:** ^1^ Florida Atlantic University, Davie, FL, USA; ^2^ 1Florida Alzheimer's Disease Research Center, Miami, FL, USA; ^3^ Mount Sinai Medical Center, Miami Beach, FL, USA; ^4^ Department of Clinical and Health Psychology (B.M.A.), University of Florida, Gainesville, FL, USA; ^5^ 1Florida Alzheimer's Disease Research Center, Gainesville, FL, USA; ^6^ University of Florida Center for Cognitive Aging and Memory, Gainesville, FL, USA; ^7^ University of Miami School of Medicine, Miami, FL, USA; ^8^ University of Florida, Gainesville, FL, USA; ^9^ Florida Alzheimer's Disease Research Center, Gainesville, FL, USA; ^10^ Wien Center for Alzheimer's Disease and Memory Disorders, Miami Beach, FL, USA

## Abstract

**Background:**

The severity of white matter hyperintensities (WMHs) is known to be linked to Alzheimer's disease (AD) and vascular dementia. Ethno‐racial differences in how WMH volumes relate to cardiovascular (CV) risk factors or AD pathology have not yet been shown. Only 30% of U.S. studies have compared WMH severity between ethnic groups, with Hispanics included in only 17%. We analyzed the contributions of demographic, cultural, CV risk factors, and plasma and brain AD biomarkers to the severity of WMHs in Hispanics and non‐Hispanic Whites (Non‐HW).

**Methods:**

Participants were from the 1Florida ADRC study, including 252 Hispanics and 172 Non‐HW (No Cognitive Impairment = 63, Non‐Amnestic Mild Cognitive Impairment = 49, Amnestic MCI = 233, Dementia = 79). Linear regression models used a quantified visual rating of MRI WMH volume as the dependent variable. Independent variables included ethnicity, age, sex, education level, CV factors (see bottom of Table 1 for listing these factors), hippocampal and cortical atrophy, amyloid PET status, and plasma *p*‐tau217. The Hispanic group also included Language proficiency and acculturation as individual predictors.

**Results:**

No significant differences were observed in CV risk factors between ethnic groups (Table 1). In the entire group, individual predictors of higher WMH scores included older age, positive amyloid PET status, *p*‐tau217, severity of hippocampal and diffuse cortical atrophy, cognitive diagnosis, and higher CV risk scores (Table 2). Ethnicity, education, and sex were non‐significant. In the multiple regression model for the total sample, only age, hippocampal atrophy, and diffuse cortical atrophy remained significant predictors (Table 3). In the Hispanic group, only diffuse cortical atrophy was associated with WMH (language proficiency and acculturation were not significant predictors in the bivariate regression models; therefore, they were not included in the multiple regression model). Adjusted R^2^ values were 0.153 for the whole sample and 0.150 for Hispanics.

**Conclusion:**

The strongest predictors of WMHs were age and measures of atrophy but not cardiovascular risk. No differences in WMHs or CV risk factors were found between WH and NHW. Results suggest that WMHs are associated with neurodegeneration, regardless of ethnicity.